# Supplementary material for: Gut microbiome composition and strain-sharing in multiplex autism spectrum disorder families
Source: Nat Commun. 2026 Feb 26;17:3255. doi: 10.1038/s41467-026-70142-7 (PMC13062010; doi:10.1038/s41467-026-70142-7)
Supplement: Supplementary file 1 — Supplementary Information [file 41467_2026_70142_MOESM1_ESM.pdf]

Description of Supplementary Figures

Supplementary Figure 1: Principal component analysis (PCA) of dietary daily intake among individuals from different groups.

Supplementary Figure 2: Differential bacterial species associated with ASD in different family types.

Supplementary Figure 3: Strain-sharing thresholds for prevalent gut bacterial species.

Supplementary Figure 4. Gut microbiome strain-sharing among individuals.

Supplementary Figure 5. Microbiome–clinical phenotype associations.

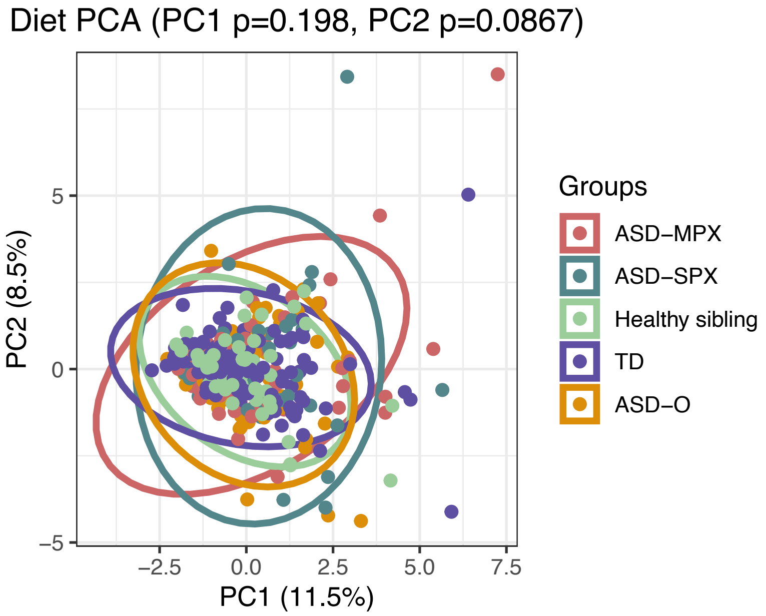

Supplementary Figure 1. Principal component analysis (PCA) of dietary daily intake among individuals from different groups. Dietary patterns assessed by daily food consumption. Kruskal–Wallis test, PC1, p =0.198. PC2, p=0.0867.

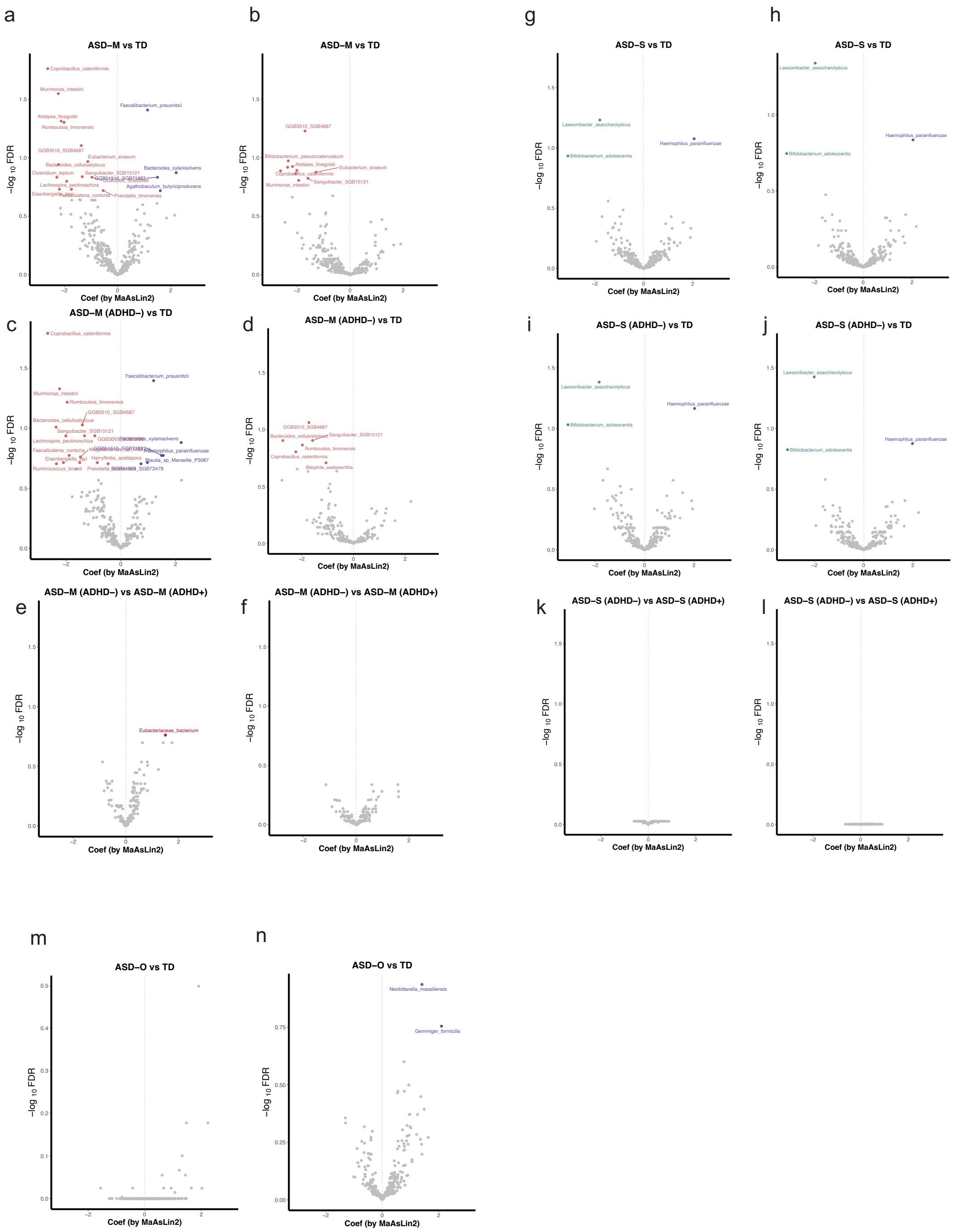

Supplementary Figure 2. Differential bacterial species associated with ASD in different family types. Associations between gut microbiota and children from different family types (ASD-M, ASD-S, ASD-O, TD) were assessed using multivariate linear models (MaAsLin2; significance: $p < 0.05$, FDR < 0.2). (a), (c), (e), (g), (i), (k), (m): Comparisons of ASD family types versus TD, and stratified analyses within the same family type by ADHD status (+/-), adjusting for confounders except dietary factors; (b), (d), (f), (h), (j), (l), (n): The same comparisons, adjusting for confounders including dietary factors.

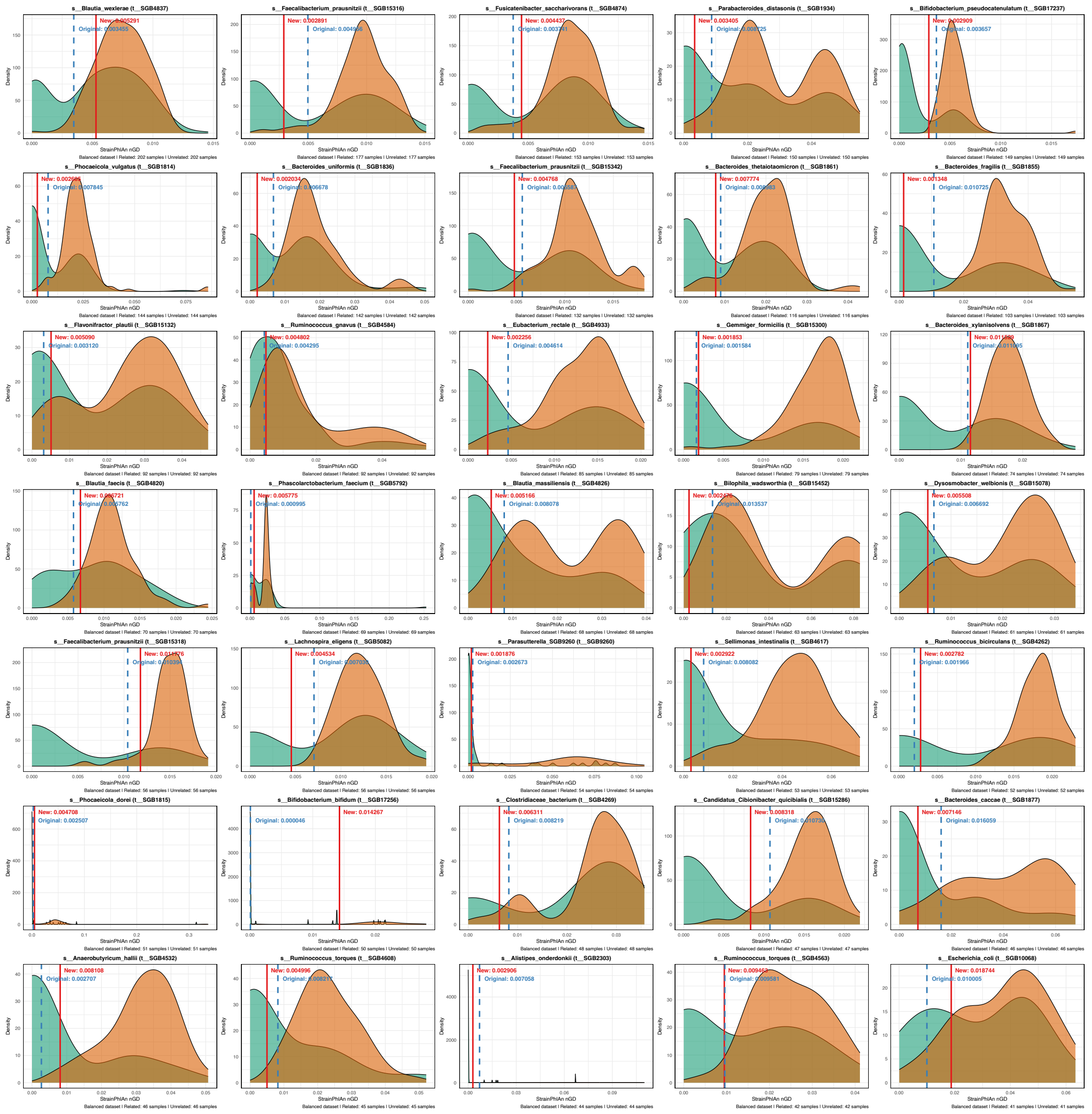

Supplementary Figure 3. Strain-sharing thresholds for prevalent gut bacterial species. Density plots of the pairwise genetic distance (nGD) for the top 35 most prevalent species-level genome bins (SGBs) in the cohort. For each SGB, the distribution of nGD between sibling pairs (likely shared strains, green) is compared to that between unrelated individuals (likely distinct strains, orange). Two strain-identity thresholds are indicated: the previously published (or 3rd-percentile) threshold (Original; blue dashed line) and the threshold optimized using Youden's index in this cohort (New; red line). Both thresholds aim to separate the two distributions, defining whether a pair of samples is considered to share the same bacterial strain.

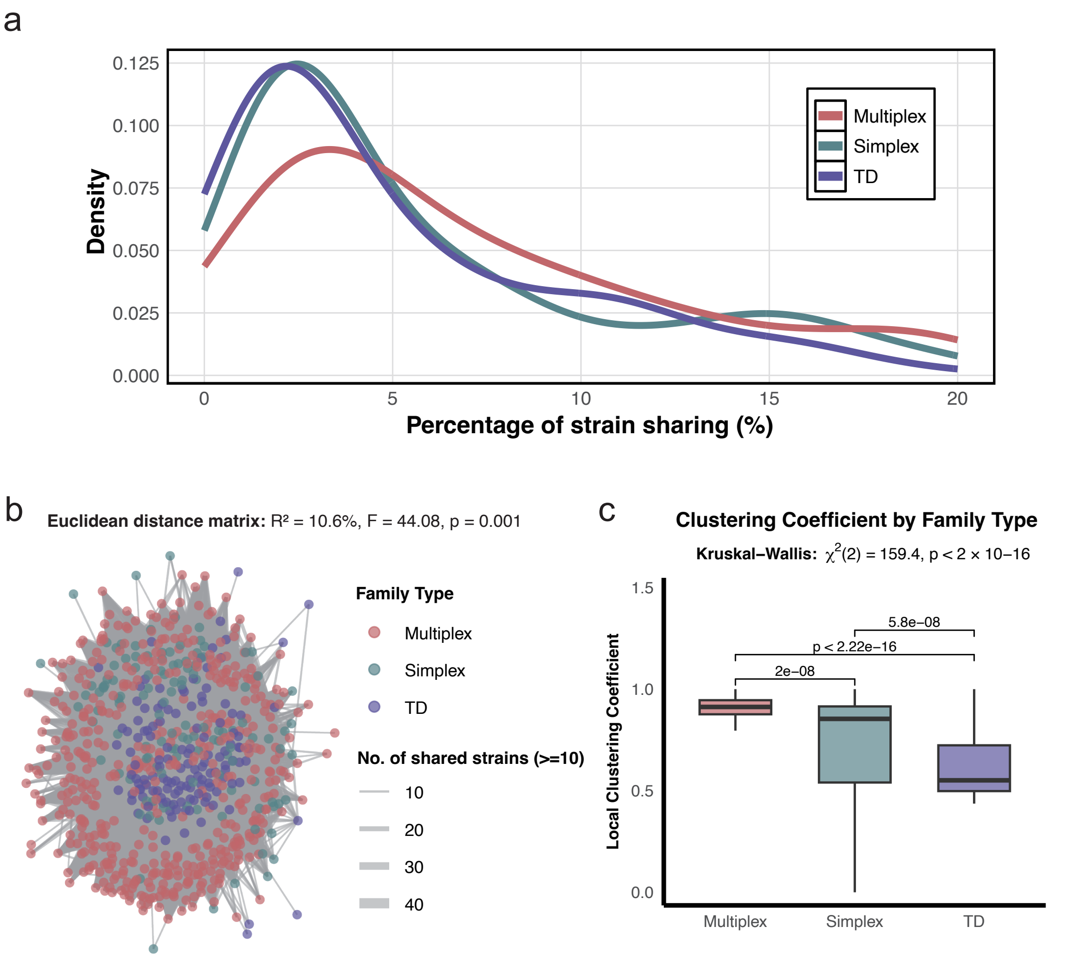

Supplementary Figure 4. Gut microbiome strain-sharing among individuals. (a) Density distributions of gut microbiome strain-sharing rates. The three curves represent comparisons within households in multiplex families (dark red), within simplex families (green), and within TD families (purple). (b) Unsupervised network of gut microbiome strain sharing among individuals within the three family types. Lines connect individuals who share microbial strains, with line width proportional to the number of shared strains. Only connections with ≥ 10 shared strains are displayed. Global differences in strain-sharing patterns across family types were assessed by PERMANOVA on a Euclidean distance matrix ($R^2 = 10.6\%$, $F = 44.08$, $p = 0.001$; based on 999 permutations). (c) Comparison of local clustering coefficients across family types. In the box plots, the centre line represents the median; box bounds indicate the 25th and 75th percentiles; whiskers extend to the minima and maxima. Kruskal–Wallis test revealed highly significant differences between groups ($\chi^2(2) = 159.4$, $p < 2\times 10{-}16$). Pairwise comparisons were performed using two-sided Wilcoxon rank-sum tests, with p values adjusted for multiple comparisons using the Benjamini–Hochberg method.

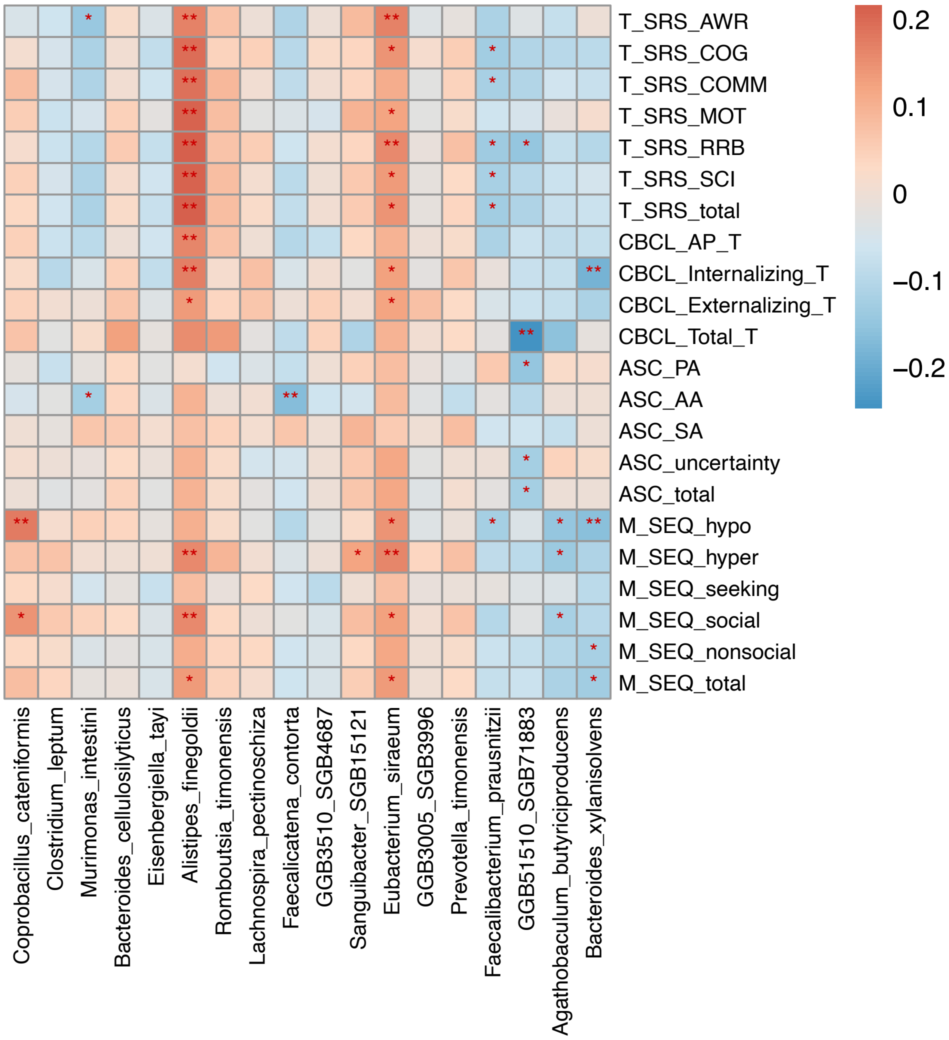

Supplementary Figure 5. Microbiome–clinical phenotype associations. Microbiome–phenotype associations for clinical parameters. Microbial species were selected by the multivariate analysis by linear models between MPX-ASD and TD (MaAsLin2; $p < 0.05$, FDR<0.2). Associations with a mark indicate significant associations (Spearman's correlation, * $p < 0.05$).
