# Supplementary material for: Gut microbiome composition and strain-sharing in multiplex autism spectrum disorder families
Source: Nat Commun. 2026 Feb 26;17:3255. doi: 10.1038/s41467-026-70142-7 (PMC13062010; doi:10.1038/s41467-026-70142-7)
Supplement: Supplementary file 2 — Description of supplementary files [file 41467_2026_70142_MOESM2_ESM.docx]

**Description of Additional Supplementary Files**

**Supplementary Data 1:** Subjects’ demographic characteristics. Statistical significance was assessed using One-way ANOVA for continuous variables and two-sided Chi-square tests for categorical variables.

**Supplementary Data 2:** Overall differences in dietary factors assessed by Kruskal-Wallis tests.

**Supplementary Data 3:** Pairwise comparisons of dietary factors using two-sided Wilcoxon rank-sum tests with Benjamini–Hochberg (FDR) correction. Generated for MaAsLin2 diet adjustment.

**Supplementary Data 4–13:** Differential bacterial species between different sub-groups. Associations were identified using two-sided multivariate association with linear models (MaAsLin2), adjusting for potential confounders including age, gender, ADHD status and dietary factors. Multiple testing correction was performed using the Benjamini–Hochberg procedure to control the False Discovery Rate (FDR). Features with an adjusted P value (Q-value) < 0.2 were considered significantly different. Supplemented for Figure 1c and Supplementary Figure 2.

**Supplementary Data 4:** ASD-M vs. TD (Adjusted: Age, Gender, ADHD).

**Supplementary Data 5:** ASD-M vs. TD (Adjusted: Age, Gender, ADHD, Diet).

**Supplementary Data 6:** ASD-M (ADHD-) vs. TD (Adjusted: Age, Gender).

**Supplementary Data 7:** ASD-M (ADHD-) vs. TD (Adjusted: Age, Gender, Diet).

**Supplementary Data 8:** ASD-M (ADHD-) vs. ASD-M (ADHD+) (Adjusted: Age, Gender).

**Supplementary Data 9:** ASD-S vs. TD (Adjusted: Age, Gender, ADHD).

**Supplementary Data 10:** ASD-S vs. TD (Adjusted: Age, Gender, ADHD, Diet).

**Supplementary Data 11:** ASD-S (ADHD-) vs. TD (Adjusted: Age, Gender).

**Supplementary Data 12:** ASD-S (ADHD-) vs. TD (Adjusted: Age, Gender, Diet).

**Supplementary Data 13:** ASD-O vs. TD (Adjusted: Age, Gender, ADHD, Diet).

**Supplementary Data 14:** Comparisons of Bray-Curtis distances between cohabiting and non-cohabiting pairs using two-sided Wilcoxon rank-sum tests with Benjamini–Hochberg correction. Supplemented for Figure 2.

**Supplementary Data 15:** Overall strain-sharing rates across family types assessed by **Kruskal-Wallis tests**. Supplemented for Figure 3.

**Supplementary Data 16:** Pairwise comparisons of strain-sharing rates using two-sided Wilcoxon rank-sum tests with FDR correction. Supplemented for Figure 3.

**Supplementary Data 17:** Post-hoc Dunn two-sided tests on gut strain sharing rates within Multiplex ASD families, Simplex ASD families and TD families. Multiple testing-corrected P values (Benjamini–Hochberg procedure) are reported in the Padj column. Supplemented for Supplementary Figure 4a.

**Supplementary Data 18:** Comparison of SGB-specific strain identity thresholds (Youden’s index vs. original methods). Supplemented for Figures 3-4 and Supplementary Figure 3.

**Supplementary Data 19:** Reproducibility assessment results for SGB-specific strain identity thresholds. Supplemented for Figures 3-4 and Supplementary Figure 3.

**Supplementary Data 20:** Association tests for shared bacterial strains using two-sided Chi-squared tests (with Yates’s correction) and two-sided Fisher’s exact tests. Supplemented for Figure 4.
