# Supplementary material for: Gut microbiome composition and strain-sharing in multiplex autism spectrum disorder families
Source: Nat Commun. 2026 Feb 26;17:3255. doi: 10.1038/s41467-026-70142-7 (PMC13062010; doi:10.1038/s41467-026-70142-7)
Supplement: Supplementary file 4 — Reporting-summary [file 41467_2026_70142_MOESM4_ESM.pdf]

Corresponding author(s): Qi Su and Siew Chien Ng

Last updated by author(s): Feb 6, 2026

Reporting Summary

Nature Portfolio wishes to improve the reproducibility of the work that we publish. This form provides structure for consistency and transparency in reporting. For further information on Nature Portfolio policies, see our Editorial Policies and the Editorial Policy Checklist.

Statistics

For all statistical analyses, confirm that the following items are present in the figure legend, table legend, main text, or Methods section.

n/a	Confirmed	
☐	☒	The exact sample size (*n*) for each experimental group/condition, given as a discrete number and unit of measurement
☐	☒	A statement on whether measurements were taken from distinct samples or whether the same sample was measured repeatedly
☐	☒	The statistical test(s) used AND whether they are one- or two-sided<br>*Only common tests should be described solely by name; describe more complex techniques in the Methods section.*
☐	☒	A description of all covariates tested
☐	☒	A description of any assumptions or corrections, such as tests of normality and adjustment for multiple comparisons
☐	☒	A full description of the statistical parameters including central tendency (e.g. means) or other basic estimates (e.g. regression coefficient) AND variation (e.g. standard deviation) or associated estimates of uncertainty (e.g. confidence intervals)
☐	☒	For null hypothesis testing, the test statistic (e.g. *F*, *t*, *r*) with confidence intervals, effect sizes, degrees of freedom and *P* value noted<br>*Give P values as exact values whenever suitable.*
☒	☐	For Bayesian analysis, information on the choice of priors and Markov chain Monte Carlo settings
☐	☒	For hierarchical and complex designs, identification of the appropriate level for tests and full reporting of outcomes
☐	☒	Estimates of effect sizes (e.g. Cohen's *d*, Pearson's *r*), indicating how they were calculated

Our web collection on statistics for biologists contains articles on many of the points above.

Software and code

Policy information about availability of computer code

Data collection	For full details see Methods; Trimmomatic (v39), Kneaddata (v0.6), MetaPhlAn4 (v4.1.4), StrainPhlAn4 (v4.1.4), GUN paralle (v2018) were used to process the microbiome sequencing data.
Data analysis	Continuous variables were expressed in median (interquartile range) whereas categorical variables were presented as numbers (percentage). Qualitative and quantitative differences between subgroups were analysed using chi-square of Fisher's exact tests for catagorical parameters and Wilcoxon rank-sum test for continuous parameters, as appropriate. The site by species counts and relative abundance tables were input into R V.4.2.2 for statistical analysis. Principal coordinate analysis (PCoA) was used to visualize the clustering of samples based on their species-level compositional profiles. Associations between diseases and microbiome composition were determined by the multivariate analysis by linear models (MaAsLin2) statistical frameworks implemented in the Huttenhower Lab Galaxy instance (http://huttenhower.sph.harvard.edu/galaxy/). Microbiota compositional characteristics, including abundance, diversity, and richness, as well as PCoA analyses, were conducted using the vegan R package V. 2.6-4. correlations between ASD clinical scores and microbial species were conducted by Spearman's correlation.

For manuscripts utilizing custom algorithms or software that are central to the research but not yet described in published literature, software must be made available to editors and reviewers. We strongly encourage code deposition in a community repository (e.g. GitHub). See the Nature Portfolio guidelines for submitting code & software for further information.

Data

Policy information about availability of data

All manuscripts must include a data availability statement. This statement should provide the following information, where applicable:

- Accession codes, unique identifiers, or web links for publicly available datasets
- A description of any restrictions on data availability
- For clinical datasets or third party data, please ensure that the statement adheres to our policy

Raw sequence data are available in the Sequence Read Archive (SRA) under BioProject accession PRJNA1377943.

Research involving human participants, their data, or biological material

Policy information about studies with human participants or human data. See also policy information about sex, gender (identity/presentation), and sexual orientation and race, ethnicity and racism.

Reporting on sex and gender	429 individuals were involved in this study. Amongst the 429 children (mean age: 7.05) studied, there were 37 multiplex ASD families (ASD children, n=75, males 74.6%, namely ASD-M), 50 simplex ASD families (ASD children, n=50; males 94%, namely ASD-S; healthy siblings, n=54, Male: 96.3%), 66 TD families (typically developing children, n=133, males 59.6%; namely TD), and 117 only-child families (ASD, n=117, males 87.1%, namely ASD-O).
Reporting on race, ethnicity, or other socially relevant groupings	All the subjects recruited in this study were Hong Kong Chinese.
Population characteristics	This is a cross-sectional and prospective cohort study involving 429 subjects from different family types in Hong Kong, China. 312 of them were from three types of multi-child families and the other 117 children were from only-child ASD families. The children with ASD were diagnosed with a formal medical certificate from a qualified psychiatrist, pediatrician, or psychologist.
Recruitment	A cohort consisting of different family types was recruited from the Child and Adolescent Psychiatric Clinic of the New Territory East Cluster (NTEC) of the Hospital Authority and the community. In addition to a self-reported diagnosis of ASD, parents were required to provide a formal medical certificate from a qualified psychiatrist, pediatrician, or psychologist confirming the ASD diagnosis before their child was included in the case group for recruitment.
Ethics oversight	The study was approved by the Joint Chinese University of Hong Kong of New Territories East Cluster Clinical Research Ethics Committee (CUHK-NTEC CREC). All subjects provided written informed consent.

Note that full information on the approval of the study protocol must also be provided in the manuscript.

Field-specific reporting

Please select the one below that is the best fit for your research. If you are not sure, read the appropriate sections before making your selection.

[x] Life sciences [ ] Behavioural & social sciences [ ] Ecological, evolutionary & environmental sciences

For a reference copy of the document with all sections, see nature.com/documents/nr-reporting-summary-flat.pdf

Life sciences study design

All studies must disclose on these points even when the disclosure is negative.

Sample size	Sample size calculation was not performed before this study. We maximized the number of eligible subjects included in this cohort to enhance the reliability of the analysis results.
Data exclusions	All 429 samples were successfully sequenced and passed the quality assessment (read depth > 10 million), thus no were excluded from the analyses.
Replication	This study was not formally replicated.
Randomization	This is an observation study without treatment groups.
Blinding	This is an observation study with no blinding as there was no treatment groups.

Reporting for specific materials, systems and methods

We require information from authors about some types of materials, experimental systems and methods used in many studies. Here, indicate whether each material, system or method listed is relevant to your study. If you are not sure if a list item applies to your research, read the appropriate section before selecting a response.

Materials & experimental systems

n/a	Involved in the study
☒	☐ Antibodies
☒	☐ Eukaryotic cell lines
☒	☐ Palaeontology and archaeology
☒	☐ Animals and other organisms
☒	☐ Clinical data
☒	☐ Dual use research of concern
☒	☐ Plants

Methods

n/a	Involved in the study
☒	☐ ChIP-seq
☒	☐ Flow cytometry
☒	☐ MRI-based neuroimaging

Plants

Seed stocks	*Report on the source of all seed stocks or other plant material used. If applicable, state the seed stock centre and catalogue number. If plant specimens were collected from the field, describe the collection location, date and sampling procedures.*
Novel plant genotypes	*Describe the methods by which all novel plant genotypes were produced. This includes those generated by transgenic approaches, gene editing, chemical/radiation-based mutagenesis and hybridization. For transgenic lines, describe the transformation method, the number of independent lines analyzed and the generation upon which experiments were performed. For gene-edited lines, describe the editor used, the endogenous sequence targeted for editing, the targeting guide RNA sequence (if applicable) and how the editor was applied.*
Authentication	*Describe any authentication procedures for each seed stock used or novel genotype generated. Describe any experiments used to assess the effect of a mutation and, where applicable, how potential secondary effects (e.g. second site T-DNA insertions, mosiacism, off-target gene editing) were examined.*
